# Traditional Medicinal Vegetables in Northern Uganda: An Ethnobotanical Survey

**DOI:** 10.1155/2021/5588196

**Published:** 2021-07-15

**Authors:** Rebecca Nakaziba, Maxson Kenneth Anyolitho, Sharon Bright Amanya, Crispin Duncan Sesaazi, Frederick Byarugaba, Jasper Ogwal-Okeng, Paul E. Alele

**Affiliations:** ^1^Faculty of Health Sciences, Lira University, Lira, Uganda; ^2^Department of Pharmacology and Therapeutics, Mbarara University of Science and Technology, Mbarara, Uganda; ^3^Department of Microbiology, Mbarara University of Science and Technology, Mbarara, Uganda

## Abstract

**Background:**

A wide range of indigenous vegetables grow in Uganda especially during rainy seasons but scarcely during droughts, except those that are commercially grown. Although a number of these vegetables have medicinal values, they have not been satisfactorily studied besides conservation. Therefore, we conducted a cross-sectional ethnobotanical survey in Northern Uganda in order to document traditional medicinal vegetables and their uses.

**Methods:**

Qualitative and quantitative approaches of data collection and analysis were employed using semistructured, interviewer-administered questionnaires as well as key informant interviews following international ethical codes. Fidelity levels and informant consensus factors were also calculated.

**Results:**

13 traditional vegetables belonging to 10 families were reported to serve as folk medicines. The most dominant families were Fabaceae (23.08%) and Solanaceae (15.38%). The most often used vegetables were *Corchorus* spp., *Hibiscus spp.*, and *Asystasiagangeticafor* musculoskeletal (51%), gastrointestinal (34.3%), and malaria (31.8%). The vegetables were cultivated in the backyard and the leaves stewed for the different ailments. The informant consensus factor was the highest for *Corchorus* spp., in the treatment of joint pain/stiffness (0.92-1) while the highest fidelity level was (60.42%) for *Amaranthus* spp., in the management of anemia.

**Conclusions:**

Northern Uganda has numerous traditional vegetables with medicinal benefits. Diseases treated range from gastrointestinal to reproductive through musculoskeletal abnormalities. The community obtains vegetable leaves from the backyard and stews them regularly for the medicinal purposes with no specific dosage. Therefore, we recommend studies to verify in laboratory models the efficacy of these vegetables and standardize the dosages.

## 1. Introduction

Despite the aggressive rivalry from conventional medicines, natural products have remained drugs of choice for some individuals due to their safety and efficacy [1]. Individuals prefer to use traditional medicines because of affordability and accessibility as well as desire for personalized health care coupled to fear for adverse events associated with synthetic drugs [2, 3]. Usage also surges when conventional medicines are ineffective in the treatment of diseases such as cancer and in the face of new infectious diseases [4, 5]. Traditional medicines of plant origin are used by about 80% of persons in the developed countries [6, 7] while more than 30% of the modern pharmacological drugs have their origin directly or indirectly linked to plants [8, 9]. An estimated 25% of the drugs prescribed worldwide are derived from plants [10] and out of the total 252 drugs in the World Health Organization's (WHO) essential medicine list, 11% are utterly of plant origin [1, 11]. Moreover, 80% of 122 plant derived drugs have their uses related to their original ethnopharmacological purposes [12].

Traditional leafy vegetables worldwide are a valuable and cheap source of nutrition for a balanced diet [13]. In addition, these vegetables serve as folk medicines [13] for treating conditions such as toothache (*Amaranthus viridis. L*.), acute abdominal pain (*Celosia argentia L.),* painful urination *(Portulacaoleracea L.*), headache (*SmithiasensitivaAit.)* and diarrhea (*C. mimosoides L.*) [13] rheumatism and cough (*Marsileaminuta Linn*), and helminthes infestation (*Spinaciaoleracea Linn.)* [14].

In Uganda, traditional vegetables are plant species which are either native or were introduced into the country a while ago and are presently being cultivated and their leaves used as a sauce to the staple foods [15, 16]. Diverse species grow in all the geographical regions of the country. However, their level of cultivation and consumption differs depending on the local customs, beliefs, practices, and staple foods of the folk as well as soil/climate types [15]. Some of these traditional vegetables have been domesticated, whereas others grow and are gathered as wild or semiwild flora [15, 16]. Domesticated vegetables are planted in home-based gardens (backyards) with trivial devotion in their production. The production of traditional vegetables is suitable for several families as they grow within a short time period shortly after the start of rains subsequent to dry seasons [15]. Further, traditional vegetables are a major source of ascorbic acid and various micronutrients in the diet [16, 17] in Uganda. The vegetables contain vitamins (A, B, and C) and proteins and minerals such as iron, calcium, phosphorus, iodine, and fluorine in varying amounts but adequate for normal growth and health [17]. According to the FAO Food Balance Sheet for Uganda, traditional food plants supply about 90% energy, 76% protein and 63% fat, and most of vitamins A and C, iron, and dietary fiber [15]. These food values are vital necessities for normal growth and defense against protein/calorie malnutrition in humans [15]. Traditional vegetables ensure a well-balanced diet in rural areas [13]. In some cases, parts of traditional vegetable species serve as staple foods such as the mature fruits of *C. maxima* and the tubers of *C. benghalensis, Ipomoea* spp.*, M. esculenta*, and *S. edule.*

Not only are these traditional vegetables a source of food, they are as well used for medicinal purposes. For example, prevention of blindness especially in children using vitamin A found in all dark green leafy traditional vegetables such as *Amaranthus* (dodo), *Solanumaethiopicum* (Nakati), *Manihotesculenta* (cassava leaves), and *Ipomeabatatas* (sweet potato leaves). On the other hand, vegetables like *Solanumindicum* subsp. *distichum* (Katunkuma) are believed to control high blood pressure [17]. In addition, the leaves of *B. pilosa* are used for wounds and boils; while the juice, for various eye and ear problems; and a decoction for rheumatism, stomach disorders, and intestinal worms; yet the roots, for malaria treatment. Other important medicinal traditional vegetables include *C. obtusifolia, Celosia argentea*, *C. benghalensis*, *Corchorus* spp., *G. abyssinica*, *Hibiscus spp.*, *L. siceraria, L. cylindrica, S. indicum*, *S. indicum* subsp. *distichum*, *T. indica*, and *Tribulus* spp. [15]. According to a study carried out at Mwana Mugimu nutrition services, traditional vegetables were identified as a critical nutritional resource (especially in children) [18]. The study suggested that families should make nutritious foods for young babies using locally available foods, including traditional vegetables in the fight against malnutrition [15]. Traditional vegetables are also used to obtain various other products such as ornaments, dyes, tobacco and coffee substitutes, pipes, ropes, sacks, mats, containers, ladles, industrial oils including drug sponges, carriers, soil fertilizers, and livestock feeds [15].

Whereas these traditional vegetables are easily accessible to the communities and would conveniently and cheaply be used in management of various disease conditions, studies regarding their medicinal uses are scanty in the country. Besides, there is poor and inadequate documentation of the traditional medicinal uses of most of these plants since it is often privately and verbally passed on from one generation to another. This leads to high risk of loss of information about these plants including their medicinal values [19, 20].Therefore, in this study, we set out to document the traditional vegetables with their medicinal uses in Northern Uganda through an ethnobotanical survey.

## 2. Methods

### 2.1. Study Site and Setting

**Figure 1:**
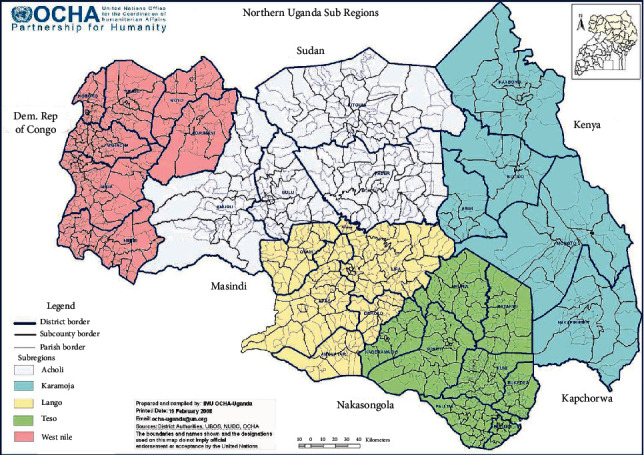
Map of Northern Uganda.

Data was collected from the Lango subregion, Northern Uganda. Northern Uganda as a region is divided into 5 subregions: Acholi, Karamoja, Lango, West Nile, and Teso. There are several ethnic groups in the region such as Acholi, Langi, and Ateso tribes. The region has a hot climate, and the natives are subsistence farmers. They mostly grow maize, soya beans, simsim, cassava, millet, ground nuts, and beans. The residents typically eat starchy foods that frequently accompanied by pasted green leafy vegetables of different kinds. They are fond of using plants including vegetables as traditional medicines for disease treatment. For instance, they use *Hibiscus spp* for the treatment of cough and the roots *Cleome gynandra* to facilitate birthing. The northern region of Uganda has 30 districts with a total population of 7,188,139 and a total area of 85,391.7 km^2^ [21] (Figure 1).

### 2.2. Study Design and Sampling

A descriptive mixed method employing both quantitative and qualitative approaches of data collection and analysis was used to describe the traditional medicinal vegetables in Northern Uganda in an ethnobotanical survey [22, 23]. This was done to enable comprehensive data collection. A multistage simple random sampling technique [24]was used to select the units (i.e., subregion, district, subcounties, parishes, and villages) for quantitative data in order to properly portray the study area and be able to generalize the study outcomes. The sample units were selected by listing the names of all units (at each stage) on small pieces of paper which were mixed up. A piece was picked, its name noted down in a book and replaced in the pool. The process was repeated until all the units were identified. One subregion, one district, four subcounties, 2 parishes per subcounty and 6 villages from each parish, and finally 5 households per village were selected. The study participants were selected based on the convenience sampling technique [24] for easy access. A sample size of 246 households (one person per household) was determined following a formula by methodology [25]. However, two [2] of the questionnaires were invalid leaving a total of 244 which are reported in this paper. Of these, five herbalists were selected using purposive and snowball techniques [24] for qualitative data.

### 2.3. Ethnobotanical Data Collection

Quantitative and qualitative data was collected using a semistructured, interviewer-administered, questionnaires [26, 27] and key informant interviews [28], respectively. Interviews were conducted in the local language (Luo) using research assistants who were skilled undergraduates from the region [26]. The data collection tool was designed to obtain details regarding the subcounty, parish, and village name; participant biodata; commonly consumed vegetables (local names); vegetables with medicinal benefits; their therapeutic uses; plant part used; style of preparation; route of administration; and quantity used [27]. In addition, the participants were requested to mention the medicinal vegetables they most commonly used, the most effective (in their opinion), and the source of information regarding the medicinal value. This information was carefully recorded in the tool during the interviews. The data collection tool was pretested before use [29] to ensure content validity, and the questionnaires were properly checked for completeness and correctness before leaving the field following data collection. A total of 246 persons were interviewed but during analysis, two were invalid. Therefore, 244 are reported in this paper. Of these, 239 (165 female and 74 male) were community members while 5 (1 female and 4 males) were known herbalists (key informants). The herbalists were individually interviewed following a key informant interview guide generated for the study [28]. The study participants were natives aged 45 years and above except for the key informants whose age was not regarded. Before conducting the interviews, the local area leaders were contacted to obtain permission for the study, and informed consent was obtained from each participant. In addition, international ethical codes of conduct were ensured throughout the study [30]. Further, the study was approved by Research and Ethics Committee (REC-_MUREC 1/7_) of Mbarara University of Science and Technology as well as the Uganda National Council for Science and Technology (UNCST-HS2589). The scientific names were obtained from previous studies in the study location [15, 31] with some of the samples identified by a botanist at Makerere University, Botany Department.

### 2.4. Data Analysis

The quantitative study responses obtained from the survey were coded and double entered into SPSS v.20 for a descriptive statistical analysis of frequencies and percentages. This was done in order to assess the significance of the vegetables in the study area. The information was summarized and reported in the form of figures and tables. Further, the informant consensus factor (ICF) was calculated to describe the effectiveness of the vegetable for each disease [32, 33]using the formula: ICF = (*n* − nt)/(*n* − 1), where *n* is the number of individual reports of a plant use for a particular illness while nt is the total number of species used by all informants for this illness. Furthermore, the fidelity level FL for the 10 commonly used vegetables for medicinal benefits was calculated as follows: FL = (*I*_*p*_/*I*_*u*_) × 100%, where *I*_*p*_ is the number of informants who suggested the use of a species for the same major use (therapeutic), and *I*_*u*_ is the total number of informants who mentioned the plant species for any use [33]. There was no major difference between the reports of the key informants and the general community. Therefore, the information obtained from the key informants was incorporated in that of the general community and reported as a whole.

## 3. Results

### 3.1. Participant Sociodemographics

A total of 244 participants' responses were valid in the current study. 239 were community members while 5 were herbalists. Majority (59.8%) were aged 45-49; 68% were females; 96.3% belonged to the Lango tribe; 56.9% were Roman Catholics; 51.6% had primary level education; while 91.4% were subsistance farmers [Table 1].

### 3.2. Traditional Medicinal Vegetables and Their Uses in Northern Uganda

13traditional vegetables, namely, *Hibiscus spp*, *Cleome gynandra, Corchorus spp*, *Crotalaria ochroleuca, Vigna unguiculata*, *Brassica oleracea*, *Cucurbita maxima D*, *Amaranthus* spp., *Capsicum spp., Solarium nigrum L., Acalypha bipartite M.*, *Cassia obtusifolia L*., and *Crassocephalumrubens*, were reported as folk medicines. They belonged to 10 families including Malvaceae (7.69%), Cleomaceae(7.69%), Tiliaceae (7.69%), Fabaceae (23.08%), Brassicaceae (7.69%), Cucurbitaceae (7.69%), Amaranthoideae (7.69%), Solanaceae (15.38%), Euphorbiaceae (7.67%), and Asteraceae (7.69%) [Table 2].

### 3.3. Vegetables Most Often Used for Traditional Medicinal Purposes

**Figure 2:**
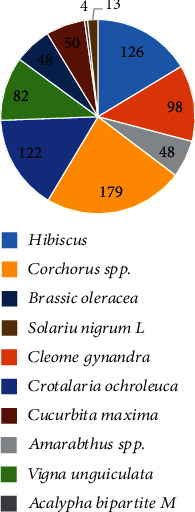
Vegetables most often used for medicinal purposes.

Out of the 13 vegetables used for medicinal purposes in the region, the most often used as reported by the participants were *Corchorus spp* (24%), *Hibiscus spp* (17%), and *Crotalaria ochroleuca* (16%) (Figure 2).

### 3.4. Most Effective Medicinal Vegetables

**Figure 3:**
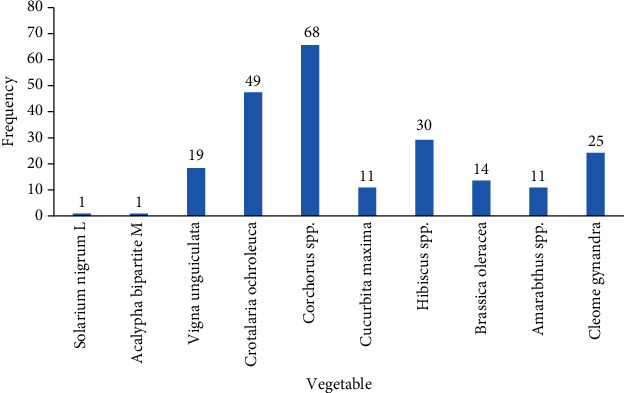
The most effective medicinal vegetables in Northern Uganda.

Reports on the most effective medicinal vegetable by the study participants indicated *Corchorus spp* (Figure 3).

### 3.5. Plant Part Used and Method of Preparation

For all of the medicinal vegetables, the leaves (>95%) were stewed (>98%). The leaves and/or young shoots are harvested, chopped into small pieces, and boiled. Groundnuts/simsim paste often added. Sometimes, the paste is not added. This is done to improve effectiveness of the vegetable in the disease condition being treated. In most cases, the sauce is eaten as a whole. In some of the conditions, only the soup is drunk. In a few instances, however, raw leaves were chewed, for example, *Acalypha bipartite M* and *Crotalaria ochroleuca* in the treatment of tooth decay (0.8%) and malaria respectively. The roots plus the stem of *Cleome gynandra* were also crushed raw and the juice obtained used in prolonged labor and placental expulsion (3.4%). In addition, the leaves of *Hibiscus spp.* were heated and placed on the wounds for healing purposes (0.4%).

### 3.6. Mode of Administration

The most applied route of administration was oral (99%). For eye/ear infections as well as toothaches, administration was topical (Table 2).

### 3.7. Cultivation of Medicinal Vegetables in Northern Uganda

**Figure 4:**
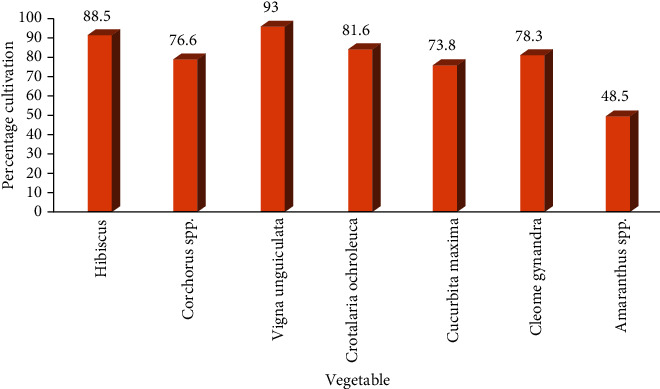
Cultivation of medicinal vegetables in Northern Uganda.

Most of the medicinal vegetables in the current study were cultivated in the backyard (Figure 4).

### 3.8. Informant Consensus Factor (ICF)

Using the reports of the study participants, the ICF for the 8 most commonly used traditional medicinal vegetable was calculated in order to highlight species that have healing potential for specific major purposes based on the homogeneity of informant's knowledge. The highest ICF value was 1 for *Corchorus* spp. (joint stiffness), *Hibiscus spp.* (poor lactation), and *Brassica oleracea*(cancer) (Table 3). Values close to 1 indicate a high rate of informant agreement on a plant.

### 3.9. Fidelity Level (FL)

The FL for the traditional medicinal vegetables which treated diseases with ICF values 0.5 and above was also calculated. According to the findings, the highest fidelity level value was 60.42% (Table 4).

## 4. Diseases Treated per Body Systems

The traditional medicinal vegetables were used to treat diseases associated with diverse body systems. The disease treated was categorized into 10 categories as indicated in Table 5.

### 4.1. Source of Information

According to our findings, the study participants obtained information regarding traditional medicinal uses of the vegetables from (1) parents/guardians (69.5%), (2) friends (23%), (3) relatives (13.8%), (4) Radio (15.9%), and (5) neighbor (7.1%). Other sources included experience (13.4%), church (0.8%), and market (2.9%).

## 5. Discussion

Not only are traditional vegetables useful as food sources, they also provide a wide range of medicinal benefits. In our study, the participants were required to mention the vegetable, conditions treated, parts used, modes of preparation and administrations, and amount. 13 vegetables were reported to be used as traditional medicines (Table 2). The most mentioned were *Corchorus spp.* (77.4%), *Hibiscus spp.* (59.8%), *Cleome gynandra* (47.3%), and *Crotalaria ochroleuca* (55.2%). *Corchorus spp*. was reported the most effective in this study (Figure 3). They were used for treating conditions which ranged from gastrointestinal complications such as abdominal pains and oral thrush through reproductive abnormalities like difficulty birthing and male sexual complications to musculoskeletal disturbances such as joint pain and stiffness (Table 2). Meanwhile, the most commonly used parts included leaves which were stewed for the medicinal applications with no specific dosage for most of the conditions treated (Table 2). Some of the vegetables were administered a number of times per day while others per week or as required (Table 2). The most commonly used and effective traditional medicinal vegetables were often cultivated especially in the backyard (Figure 4). Most of the participants obtained information regarding the medicinal uses of the traditional vegetables from their parents or guardians. Some of the traditional vegetables' medicinal applications documented in the current study relate to earlier findings [15] but a number of them do not. For instance, *Hibiscus* spp. was used for poor appetite, nausea, low saliva secretion, anemia, postpartum abdominal pain, poor lactation, oral thrush, skin swellings, wounds, ulcers, body swellings –esp. stomach swellings, poor vision, mouth sores with pus, cough, cold, flu, toothache, bone strength, painful eyes, and poisoning in the current study. These findings agree with those of Qi and Aziz [34, 35] in which the plant was found to treat sores and wounds, along with the findings of Mahadevan and Kamali [36, 37] where the plant was found to be useful as an antihelminth, antibacterial, and for cough. In addition, *Hibiscus spp.* is reported to be lactogenic [38, 39], in agreement with the current study. *Cleome gynandra* was used in the management of poor appetite, abdominal pain, scorpion bite, ringworm, difficult/prolonged labor, removal of retained placenta, postpartum bleeding, extreme headache, worm infestation, and eye/ear infections including removal of blood clots. These findings could be explained by the antimicrobial activity of the plant as reported by Ajayiyoeba and Amanirampa [40, 41] where the plant was reported to exhibit antibacterial and antifungal activity. In addition, Scippers and Kamatenesi [42, 43] found *Cleome gynandra* useful in migraine headaches, ear infections, and abdominal pains coupled to acceleration of labor and reduction of postpartum hemorrhage just as the current study findings. *Corchorus* spp. was used to treat joint pain and stiffness as well as weak joints. It was also found to strengthen bones and thus prevent fractures as well enhance fracture healing. This could be attributed to the fact that the plant is rich in calcium as reported by Idris [44] which favors mineralization thus strengthening the bones or due to the antioxidant activity of the plant which activates differentiation of osteoblasts, enhances bone mineralization, and reduces osteoclast activity [45, 46]. In Zimbabwe, *Corchorus spp.* is used for backaches [47] which is in agreement with the current study findings since the study participants reported using the plant for body aches. On the contrary, *Corchorus spp* is used in Benin for cardiac insufficiency, fever, malaria, female fertility, ulcerations, and gastrointestinal problems [48]. The plant was also reported to be useful as an antiulcer, laxative/purgative in the current study probably due to its richness in fiber [49], and its gastroprotective effects [50, 51]. *Crotalaria ochroleuca* was found by the current study to treat malaria, abdominal pain, ulcers, epilepsy, chest pain, body aches, hypertension, and diabetes (Table 2). These findings agree with those of Anywar and Ashuraduzzaman [31, 52] where the plant was found to treat malaria and relieve bronchospasms which could be responsible for the chest pain in the current study. According to a study conducted in Nigeria, the plant was found to have antibacterial and antifungal activity [53]. This could explain its use for abdominal pains, Brucella, cough, and fever in the current study. *Vigna unguiculata* was reported to alleviate poor appetite, abdominal pains, ulcers, and visual impairment in the current study. The findings of Kritzinger et al. and Sayeed et al indicated that the plant had antimicrobial activity [54] [55]. These findings support the current use of the plant for abdominal pains. In addition, this is a green leafy vegetable rich in vitamin A which is well known for improving sight [56]. *Brassica oleracea* was used for ulcers, hypertension, malaria, constipation, epilepsy, and sore throat in the current study. This could be partly explained by the fact that the plant is bioactive [57] and fiber rich [58]. *Cucurbita maxima* was found to improve male sexual activity and fetal health, enhance wound healing, enhance memory, and treat hepatitis B and coronary artery disease in the present study. On the contrary, a study by Dubey showed that the plant was used as a remedy for tape worms, as a sedative, a tonic, a diuretic, has anticancer, antidiabetic, and hepatoprotective activity [59]. The plant was found by Solomon et al. to have antimicrobial activity [60] justifying the wound healing effect in the current study. *Amaranthus* spp. has been reported to boost blood levels [61] while *Crotalaria ochroleuca* as an antimalarial agent [31]. These findings coincide with the current reports. The most frequent plant part used in the current study was the leaves. This was in agreement with other related studies [13, 62, 63]. The informant consensus factors (ICF) were calculated for the most commonly used traditional medicinal vegetables to ascertain the consistency of informants' ethnopharmacological knowledge (table 3). Usage of a variety of vegetables for a particular disease greatly reduced the ICF while for conditions where only a few vegetables were used, the resultant ICF was higher. High ICF values indicated wide usage (informant agreement) of a vegetable for a particular disease and hence calling for further pharmacological and phytochemical investigations. The vegetable and conditions with the highest ICF were *Corchorus spp.* for joint stiffness, joint weakness, and pain (ICF = 1); *Hibiscus spp.* for poor lactation; *Crotalaria ochroleuca* for malaria and body aches (ICF > 0.83); and *Cleome gynandra* for ringworm and abdominal pain (ICF > 0.75). The high ICF for *Corchorus* spp. contradicts findings of other studies within and without the region [64–68]. Thus, the uses in the current study (joint pain/stiffness) differ from the uses elsewhere (muscle spasms, wounds). The ICF findings for *Hibiscus spp.* and Crotalaria *ochroleuca* as well as for *Cleome gynandra* agree with other studies in the region [31, 39–41]. On the other hand, the fidelity levels (FL) for vegetables with ICF values ≥ 0.5 were calculated to quantify their importance to treat a disease (Table 4). The FL values were the highest for *Amaranthus spp*. (Anemia, 60.4%) and *Crotalaria ochroleuca* (malaria, 57.6%). This is supported by previous findings in the country [31, 61]. However, the current findings for *Hibiscus* spp. (poor appetite, 51.8%) and *Corchorus spp*. (joint pain and stiffness, 43.8%) contradict previous studies in other regions [39, 68].High FL values indicate a high cultural significance for the vegetable. In a bid to strengthen conservation, several ethnobotanical studies are being conducted in the country [64, 65, 69]. However, these studies major on documentation of medicinal plants and their uses rather than engaging the communities to actively participate in the conservation process at family levels. As such, community sensitization with these studies' findings is highly called for if these medicinal plants are to be conserved and preserved for the generations to come.

## 6. Conclusion

Northern Uganda has numerous traditional vegetables with medicinal benefits. Diseases treated range from gastrointestinal to reproductive through musculoskeletal abnormalities. The community obtains vegetable leaves from the backyard and stews them regularly for the medicinal purposes with no specific dosage. Therefore, we recommend studies to verify in laboratory models the efficacy of these vegetables and standardize the dosages.

## Figures and Tables

**Table 1:** Participants' sociodemographic profile.

Variable	Description	Frequency	Percentage
Age	45-49 years	146	59.8
	50-54 years	38	15.6
	55-59 years	18	7.4
	60 and above	42	17.2

Gender	Female	166	68.0
	Male	78	32.0

Tribe	Lango	235	96.3
	Acholi	6	2.5
	Alur	1	0.4
	Bantu	2	0.8

Religious affiliation	Anglican	77	31.8
	Roman Catholic	139	56.9
	Moslem	2	0.8
	Pentecostal	25	10.5
	Other	1	0.4

Education level	Informal	77	31.6
	Primary	128	52.5
	Secondary	35	14.3
	Other	4	1.6

Source of income	Subsistence farming	223	91.4
	Business	12	5.0
	Formal employment	6	2.5
	Other	3	1.2

**Table 2:** Traditional medicinal vegetables in Northern Uganda.

Vegetable (local name/scientific/family name)	Diseases treated	Plant part used	Mode of preparation, administration
Amalakwang/*Hibiscus spp/*Malvaceae	Poor appetite (31%)	Leaves	Stewed or soup drunk, 2× a day or week
	Nausea (0.8%)	Leaves	Stewed without extracting soup 2× daily
	Low saliva secretion (0.4%)	Leaves	Stewed as above once a week
	Low blood level (1.7%)	Leaves	Stewed (but not pasted for better results) once a day
	Sickle cell disease (0.8%)	Leaves	Stewed regularly
	Post-partum abdominal pain (0.4%)	Leaves	Stewed once a day
	Low milk production during lactation (10.8%)	Leaves/seeds	Stewed 3× a day for 1 week after delivery while seeds are roasted, ground, and eaten 3× a week
	Oral thrush (0.8%)	Leaves	Stewed 2× daily for a week
	Wounds (0.4%)	Leaves	Roasted/heated and placed on the wound 2× daily until recovery
	Malaria (1.7%)	Leaves	Stewed 2× daily for 3 days
	Ulcers (2.1%)	Leaves/seeds	Leaves stewed 2-3 times a week lifelong while seeds are grounded and mixed with other foods regularly
	Body swellings–esp. stomach swellings (1.3%)	Leaves	Roasted and rubbed on the affected part regularly until recovery Mixed with apuruk, boiled, and soup drunk 2× a day
	Poor vision (1.7%)	Leaves	Stewed 2× a day
	Mouth sores with pus (0.8%)	Leaves	Rough surface of raw leaves used to scrub sores until cleared
	Cough (2.1%)	Leaves/roots	Leaves may be stewed or 3-4 raw leaves chewed 2× a day while 2-3 raw roots can also be chewed
	Cold, flu (0.4%)	Leaves	Stewed as required
	Cannibalism (0.4%)	Leaves	Boiled together with other herbs and eaten once a day for 1 week
	Toothache (0.4%)	Leaves	Half boiled and placed on gum as required
	Bone strength (1.3%)	Seeds	Dried, fried, pounded, and stewed mixed with other foods
	Waist pain (0.8%)		
	Painful eyes (0.4%)	Leaves	Crushed to obtain juice and dropped into the eye 2× daily
	Poisoning (0.8%)	Leaves	Boiled–without salt and eaten or soup drunk 3× a day until recovery

Akeo/*Cleome gynandra/*Cleomaceae	Poor appetite (4.6%)	Leaves	Stewed 2× a week
	Bloating (0.4%)	Leaves	Stewed once a week
	Abdominal pain (14.2%)	Root/leaves+ stem	Raw roots are chewed or pounded, juice extracted, and drunk 3× daily for 3days or roasted, stewed, and eaten at the time of pain while raw leaves are chewed or stewed 1-3× a day/3× a week; leaves also boiled, soup extracted, and drunk 3× a day for 4days
	Constipation (0.4%)	Leaves	Stewed regularly
	Ring worm+ skin rashes (9.6%)	Leaves	Crushed and applied (rubbed) on the affected area 2-3× a day for 1 week or until recovery
	Improve sexual activity in men (0.4%)	Leaves	Stewed regularly
	Extreme headache (3.3%)	Leaves	Pound, tied in a cloth, and wrapped around the head for 1 hour twice a day or cooked, soup drained, and eaten 3× a week
	Hypertension (0.4%)	Leaves	Stewed for one month
	Eye infection (1.7%)	Leaves	Crushed to obtain juice which is applied to the eye once during infection or 2× a day for 3 days
	Painful eyes (0.4%)	Leaves	Rubbed and placed closer to the eyes for the vapor to enter, 3× a day
	Otitis media (0.4%)	Roots	Pounded, water added, and filtered and dropped in ear 2× a day
	Removing blood clots from eyes (0.4%)	Leaves+ stem	Stewed alone and eaten 3× a day
	Worm infestation (1.3%)	Leaves	Crushed and the juice rubbed on affected area once a day for 2 weeks
	Visual impairment (4.2%)	Leaves/roots	Leaves stewed 3× a day/week while roots are pounded, juice extracted, and drunk 3× a day
	Malaria (5%)	Leaves	Raw leaves chewed 3× a week or boiled, soup extracted, and drunk 3× a day for 3 days
	Diabetes (0.4%)	Leaves	Stewed daily
	Peptic ulcers (0.8%)	Leaves	Boiled, soup removed, and eaten 3× a day
	Difficulty in delivery (0.8%)	Root	Raw roots chewed once a day
	Prolonged labor (0.8%)	Leaves	Boil, juice extracted, mixed with tea leaves, and drunk once
	Removal of placenta after delivery (0.8%)	Leaves +stem +roots	Raw–washed and crushed to obtain juice and drunk in small quantities frequently
	Postpartum abdominal pain (0.5%)	Leaves + stem	Stewed, pasted and eaten 3× a day
	Miscarriages (0.4%)	Leaves	Stewed regularly
	Sickle cell (0.4%)	Leaves/seeds	Leaves stewed and mixed with avocado while seeds are pounded, water added, juice removed, and drunk (~150 ml) 3× a day
	Fever–in children (0.8%)	Leaves	Crushed, mixed with water and the child bathed 3× a day
	Scorpion bite (1.6%)	Leaves	Cooked and soup removed and drunk for 3 days
	Toothache (0.8%)	Roots	Crushed to obtain juice which is applied to teeth 2× a day for 3days

Otigo/*Corchorus spp/*Tiliaceae	Joint pain and stiffness (33.9%)	Leaves/seeds	Leaves stewed (alone for better results) and eaten regularly while seeds are stewed with other foods 2-3× a week lifelong and frequently for HIV patients
	Waist pain during menstruation (0.4%)	Leaves	Stewed (+/- paste) regularly
	Prevent bone fracture in case of accident (1.3%)	Leaves	Stewed (+/-other foods) 2× a day
	Joint lubrication and strength (9.2%)	Leaves	Stewed regularly
	Heartburn (0.8%)	Leaves	Raw or stewed (but not pasted) 2× a day
	Poor appetite (10.9%)	Leaves	Stewed, 1-2 a day/week
	Ulcers (2.5%)	Leaves/seeds	Leaves stewed 2× a day for 2 weeks while seeds grounded and mixed with other foods/also as tea 2× a day for 1 week
	Purgation (2.5%)	Leaves/fruits	Stewed 2-3× a day
	Flatulence (0.4%)	Leaves	Stewed regularly
	Bone pains (3.7%)		
	Fasten fracture healing (1.3%)	Leaves	Cooked + silver fish
	Muscle rigidity (contractures) (0.8%)	Leaves	Stewed 2× daily
	Weak muscles (0.4%)	Leaves	Stewed 3× a day
	Engorged blood vessels (0.4%)	Leaves	Stewed 3× daily
	Constipation (5.9%)	Seeds	Cooked and eaten once after constipation or twice a week
	Malnutrition (0.8%)	Leaves	Stewed and pasted, 2× a day
	Scabies (0.8%)	Leaves	Dried, pounded, mixed with petroleum jelly, and applied to the skin 2× a day
	Anemia (1.7%)	Leaves	Stewed regularly
	Rough voice (smoothening) (0.4%)	Leaves/seeds	Stewed 3× a day
	Mental problems (0.4%)	Leaves	Stewed daily
	Poisoning (0.4%)	Leaves	Stewed 2× a week
	Sickle cell disease (0.4%)	Leaves	Stewed regularly
	Vision (0.4%)	Leaves/seeds	Stewed daily
	Hemorrhoids (0.4%)	Seeds	Stewed regularly
	Abdominal pain (0.4%)	Leaves	Stewed as required
	Enhance recovery from sickness (0.4%)	Leaves	Stewed alone
	Improve fetal health and ease birthing (0.8%)	Leaves	Stewed alone
	Low immunity esp TB patients (0.4%)	Fruit	Stewed 2× a week
	Painful swallowing, GI obstruction (0.4%)	Leaves	Stewed as required
	Poor digestion (0.8%)	Leaves	Stewed regularly

Alaju/*Crotalaria ochroleuca/*Fabaceae	Anemia (0.8%)	Leaves	Stewed regularly
	Malaria (31.8%)	Leaves	A hand full of raw leaves chewed once a day, or leaves are boiled (not pasted) and eaten or soup drunk (children) 2-3× a day for 2-4 days
	Abdominal pain (6.7%)	Leaves	A half of a handful of raw leaves chewed 2× a day or leaves are boiled (+ salt only) 2-3× a day for 1-2days
	Chest pain (0.4%)	Leaves	Stewed daily
	Body aches (2.9%)	Leaves	Stewed without paste daily
	Visual impairment (2.5%)	Leaves	Stewed daily
	Cough (0.8%)	Leaves	Raw leaves chewed 2× daily
	Poor appetite (1.3%)	Leaves	Stewed 1-3× a day
	Ulcers (3.8%)	Leaves	Stewed 2× daily
	Heart burn (0.8%)	Leaves	Stewed regularly
	Fever (0.8%)	Leaves	Stewed as required
	Epilepsy (0.4%)	Seeds	Pounded and mixed with other herbs and drunk 2× a day for 3 days
	Headache (0.8%)	Leaves	Stewed (+ salt only) 2× a day frequently
	HIV symptoms (1.6%)	Leaves	Stewed 3× a day, 2× a week life long
	Malnutrition (1.3%)	Leaves	Boiled, soup extracted and drunk 3×during childhood
	Brucella (0.4%)	Leaves	Stewed until recovery
	Eye infections-itching (0.8%)	Leaves	Stewed 2-3× a day
	Hypertension (1.3%)	Leaves	Raw leaves chewed or stewed daily
	Diabetes (0.8%)	Leaves	Raw leaves chewed or stewed daily

Bojo/*Vigna unguiculata/*Fabaceae	Anemia (1.3%)	Leaves	Stewed regularly
	Low vitamins (7.9%)	Leaves	Stewed 4× a day or raw leaves chewed 2× a day for 2 days or 2× a week
	Poor appetite (5.9%)	Leaves	Raw/stewed 2× a week
	Visual impairment (5%)	Leaves	Stewed 4× a week regularly
	Immune boosting (3.8%)	Leaves	Stewed and pasted 2× a day
	General body weakness (0.4%)	Leaves	As above
	Hernia (0.4%)	Leaves	Stewed with Otigo regularly
	Poorlactation (1.6%)	Leaves	Stewed at least 4× a day
	Cancer (0.4%)	Leaves	Raw leaves chewed regularly for 3months
	Improve sexual activity in men (1.6%)	Leaves	Stewed
	Malaria (4.2%)	Leaves	Stewed 3× a day, 3× a week
	Appendicitis (0.4%)	Leaves	Stewed regularly
	Abdominal aches (0.4%)	Leaves	Raw leave eaten 3× a day for 2 days
	Ulcers (2.9%)	Leaves	Raw leaves chewed 2-3× a day or stewed once a day
	Wounds (0.8%)	Leaves	Crushed and applied to the wound
	Diabetes (1.7%)	Leaves	Mixed with acacia (Garcia), crushed to extract juice, and drunk 2× a month stewed (+ paste) once daily or raw leaves chopped and eaten daily

Kabici/*Brassica oleracea/*Brassicaceae	Hemorrhoids (1.7%)	Leaves	3-4 raw leaves chewed once daily for1 week
	Heart burn (2.1%)	Leaves	Stewed regularly
	Cancer (2.1%)	Leaves	Stewed twice a day
	Ulcers (10.9%)	Leaves	Half cooked + ground nuts 3× a daily
	High blood pressure (+garlic) (2.1%)	Leaves	Raw leaves chewed frequently
	Constipation (1.3%)	Leaves	Raw/half cooked eaten 2x daily
	Drowsiness (0.8%)	Leaves	Raw/half cooked eaten 2× daily
	Epilepsy (0.4%)	Leaves	Raw leaves eaten 3× a daily
	Malaria (0.4%)	Leaves	Raw leaves eaten as required
	Sore throat (0.8%)	Leaves	Stewed or raw, eaten 2× a day
	Poor appetite (1.3%)	Leaves	Stewed

Ocwica/*Cucurbita maxima/*Cucurbitaceae	Malaria (6.7%)	Leaf/seeds	Leaves stewed while seeds are roasted, coat removed, and eaten 3× daily for 3 days
	Improves health during pregnancy (0.8%)	Leaves	Stewed and pasted regularly
	Abdominal pain (1.7%)	Leaves	Stewed daily
	Hepatitis B (1.3%)	Leaves	Stewed (+ salt+ red pepper) 2×daily
	(+cabbage) coronary artery disease (0.4%)	Leaves	Raw leaves chewed 3× a day as required
	Poor vision (0.8%)	Leaves	Stewed, not pasted
	Improve sexual activity in men (0.8%)	Leaves/seeds	Stewed or raw seeds chewed 2× a day
	Poor appetite (4.6%)	Leaves/seeds	Stewed regularly
	High blood pressure (1.7%)	Seeds	Uncoated and eaten raw frequently
	Immune boosting (2.5%)	Leaves	Stewed
	Urinary tract infections (0.4%)	Leaves	Stewed 2× a day
	Memory enhancement (0.4%)	Fruits/seeds	Fruit-boiled and seeds–dried, fried, and coat removed before eating
	Ring worm (0.4%)	Leaves	Crushed, juice extracted, and applied to affected area 3× a day for 1 week

Abuga/*Amaranthus spp/*Amaranthoideae	Anemia (12.1%)	Leaves/seeds	Leaves stewed, seeds put in water, add sugar, and~300 ml drunk 1-2× a day
	Poor child growth (0.4%)	Leaves	Stewed
	Poor appetite (7.1%)	Leaves	Stewed 2× a week
	Hepatitis B (0.4%)	Leaves	Stewed 2× daily
	Malnutrition (0.4%)	Leaves	Stewed 2× a week

Pot kamalara/*Capsicum* spp./Solanaceae	Hypertension (0.8%)	Leaves	Stewed (+ paste) regularly
	Poor vision (0.8%)	Leaves/fruit	Leaves stewed regularly; ripe fruit eaten daily
	Stomach aches (1.6%)	Leaves	Stewed once a week
	Hemorrhoids (0.4%)	Leaves	Stewed regularly

Ocuga/*Solarium nigrum L/*Solanaceae	Stomach aches (1.7%)	Leaves	Stewed (+salt only) 2× a day for 4 days or raw leaves are crushed to obtain juice which is drunk (~250 ml) 3× a day
	Peptic ulcers (0.8%)	Leaves	Stewed alone 2× a day
	Skin infections (0.4%)	Leaves	Stewed once daily
	Visual problems (1.7%)	Fruit/leaves	Ripe fruit eaten once daily for 4 days while leaves are stewed 2× a day
	Malaria (2.1%)	Leaves	Stewed once a day for 3 days
	Eye infection (0.4%)	Leaves/fruits	Leaves stewed 2× a week lifelong while ripe fruits are eaten regularly
	Weak bones (0.8%)	Leaves	Stewed 2× daily
	Immune boosting (0.4%)	Leaves	Stewed regularly
	(+Ayuu) malnutrition (0.8%)	Fruit/leaves	Ripe fruit eaten regularly while leaves are half cooked and eaten 3× a day until wellbeing

Ayuu bap/*Acalypha bipartite M*/Euphorbiaceae	Tooth decay (0.8%)	Leaves	Raw leaves chewed 2× a day for 4 days
	Skin infections (0.4%)	Leaves	Pounded, allowed to dry and mixed with petroleum jelly, and applied to skin daily
	Leprosy (0.4%)	Leaves	As above
	Stomach aches (0.4%)	Leaves	Mixed with alaju and stewed 2× a day
	Diarrhea (1.3%)	Leaves	Stewed and eaten once after diarrhea
	Constipation (0.4%)	Leaves	Stewed once a week
	Facilitate growth in children (0.8%)	Leaves	Stewed regularly

Oyado/*Cassia Obtusifolia L/*Fabaceae	Diarrhea (0.4%)	Leaves	Stewed (+paste) 3× a day
	Headache (0.4%)	Leaves	Stewed 3× a week

Apuruk/*Crassocephalumrubens/*Asteraceae	Bad oral smell (0.4%)	Leaves	Stewed, soup drained, and eaten once a week
	Weak bones (0.4%)	Leaves	Stewed 2× daily

**Table 3:** ICF values for the diseases commonly treated by the traditional medicinal vegetables in Northern Uganda.

Vegetable	Condition	No of participants report on condition (*n*)	Total No. of species for condition (nt)	ICF = (*n* − nt)/(*n* − 1)
*Corchorus spp.*	Joint stiffness	67	1	1
	Constipation	14	4	0.77
	Poor appetite	26	9	0.68
	Purgation	6	1	1
	Joint pain	14	2	0.92
	Joint weakness	22	1	1
	Weak bones	7	2	0.83

*Hibiscus spp.*	Poor appetite	74	10	0.88
	Cough	5	2	0.75
	Poor lactation	26	1	1
	Ulcers	5	6	-0.25

*Crotalaria ochroleuca*	Malaria	76	8	0.91
	Body aches	7	2	0.83
	Poorhealth	5	2	0.75
	Poor vision	6	9	-0.6
	Abdominal pain	16	8	0.53
	Ulcers	9	6	0.38

*Cleome gynandra*	Malaria	12	8	0.36
	Poor vision	10	9	0.1
	Headache	8	4	0.57
	Poor appetite	11	10	0.1
	Ring worm	21	2	0.95
	Abdominal pain	34	8	0.79

*Vigna unguiculata*	Poor appetite	14	10	0.31
	Poor vision	12	9	0.27
	Immune boosting	9	4	0.63
	Malaria	10	8	0.22
	Ulcers	7	6	0.17

*Cucurbita maxima*	Malaria	16	8	0.53
	Poor appetite	11	10	0.1
	Poor health	6	3	0.6

*Amaranthus spp*.	Anemia	29	7	0.79
	Poor appetite	7	10	-0.5

*Brassica oleracea*	Heart burn	5	3	0.5
	Ulcers	26	6	0.8
	High blood pressure	5	6	0.25
	Cancer	5	1	1

**Table 4:** Fidelity levels of the most common medicinal vegetables.

Vegetable	Condition	No of participants report on condition (*I*_*p*_)	Total No. of reports for any use (*I*_*u*_)	FL = (*I*_*p*_/*I*_*u*_) × 100
*Corchorus spp*.	Joint pain and stiffness	81	185	43.78
	Constipation	14	185	7.57
	Poor appetite	26	185	14.05
	Joint weakness	22	185	11.89
	Weak bones	7	185	3.78

*Hibiscus spp*.	Poor appetite	74	143	51.75
	Cough	5	143	3.50
	Poor lactation	26	143	18.18

*Crotalaria ochroleuca*	Malaria	76	132	57.58
	Body aches	7	132	5.30
	Poor health	5	132	3.79
	Abdominal pain	16	132	12.12
	Headache	8	113	7.08
	Ring worm	21	113	18.58
	Abdominal pain	34	113	30.09

*Vigna unguiculata*	Immune boosting	9	82	10.98

*Cucurbita maxima*	Malaria	16	56	28.57
	Poor health	6	56	10.7

*Amaranthus spp*.	Anemia	29	48	60.42

*Brassica oleracea*	Hemorrhoids	4	56	7.14
	Heart burn	5	56	8.9
	Ulcers	26	56	46.43
	Cancer	5	56	8.93

**Table 5:** Diseases treated by traditional vegetables in Northern Uganda per body system.

System	Diseases treated
Digestive system	Poor appetite, nausea, low saliva production, oral thrush, peptic ulcers, abdominal pain, bloating, flatulence, purgation, heart burn, diarrhea, bad oral smell, constipation, hemorrhoids, sore throat, hernia
Reproductive system	Postpartum abdominal pain, poor lactation, sexual difficulties, prolonged labor, placenta removal, pregnancy, miscarriages
Endocrine system	Diabetes, goiter
Musculoskeletal system	Waist and backaches, joint pain and stiffness, joint weakness, bone fractures, muscle rigidity, tooth decay
Respiratory system	Cough, flu/cold
Renal system	Urinary tract infections
Cardiovascular system	Hypertension, anemia, headache, coronary artery disease, blood vessel engorgement
Nervous system	Poor vision, mental illnesses, memory enhancement, drowsiness, epilepsy
Integumentary system	Skin rashes and infections, leprosy, ring worm, scabies, wounds
Others	Malnutrition, growth retardation, eye/ear infections, immune boosting, malaria, helminth infestation, HIV symptoms, hepatitis B, wound healing, hang over, cancer, Brucella, fever, sickle cell disease, poisoning, rough voice, scorpion bite

## Data Availability

The datasets generated and/or analyzed during the current study may be obtained from the corresponding author upon reasonable request.
